# Comparison of Extraction Methods for the Detection of Tick-Borne Encephalitis Virus RNA in Goat Raw Milk and Cream Cheese

**DOI:** 10.1007/s12560-022-09535-y

**Published:** 2022-09-20

**Authors:** Irene Müller, Nadine Althof, Bernd Hoffmann, Christine Klaus, Katja Schilling-Loeffler, Alexander Falkenhagen, Reimar Johne

**Affiliations:** 1 German Federal Institute for Risk Assessment, Max-Dohrn-Straße 8-10, 10589 Berlin, Germany; 2 Institute for Diagnostic Virology, Friedrich-Loeffler-Institut, Südufer 10, 17493 Greifswald-Insel Riems, Germany; 3 Institute for Bacterial Infections and Zoonoses, Friedrich-Loeffler-Institut, Naumburger Str. 96a, 07743 Jena, Germany

**Keywords:** Tick-borne encephalitis virus, Detection method, Goat, Milk, Cheese, Internal process control

## Abstract

Infection with the tick-borne encephalitis virus (TBEV) can cause meningitis, meningoencephalitis and myelitis in humans. TBEV is an enveloped RNA virus of the family *Flaviviridae*, which is mostly transmitted via tick bites. However, transmission by consumption of virus-contaminated goat raw milk and goat raw milk products has also been described. Only a few methods have been reported for the detection of TBEV in food so far. Here, we compare different virus extraction methods for goat raw milk and goat raw milk cream cheese and subsequent detection of TBEV-RNA by RT-qPCR. Langat virus (LGTV), a naturally attenuated TBEV strain, was used for artificial contamination experiments. Mengovirus and the human coronavirus 229E were compared to assess their suitability to serve as internal process controls. Out of three tested extraction protocols for raw milk, sample centrifugation followed by direct RNA extraction from the aqueous interphase yielded the best results, with a recovery rate (RR) of 31.8 ± 4.9% for LGTV and a detection limit of 6.7 × 10^3^ LGTV genome copies/ml. Out of two methods for cream cheese, treatment of the samples with TRI Reagent® and chloroform prior to RNA extraction showed the best RR of 4.7 ± 1.6% for LGTV and a detection limit of 9.4 × 10^4^ LGTV genome copies/g. RRs of Mengovirus and LGTV were similar for both methods; therefore, Mengovirus is suggested as internal process control virus. The developed methods may be useful for screening or surveillance studies, as well as in outbreak investigations.

## Introduction

The tick-borne encephalitis virus (TBEV) belongs to the genus *Flavivirus* within the family *Flaviviridae* (ICTV, 2020). It is an enveloped virus particle with a positive-sense, single-stranded RNA genome of approximately 11 kb in length (Muhd Radzi et al., 2015; Rodrigues et al., 2019). The clinical course after TBEV infection can range from asymptomatic infections and mild febrile disease to severe fatal courses involving meningitis, meningoencephalitis and myelitis (Ruzek et al., 2019). Until now, seven TBEV subtypes have been identified which differ in virulence and geographical distribution (Deviatkin et al., 2020). The subtype TBEV-FE which is mainly present in Asia usually causes the most severe disease with case/fatality rates up to 40% (Pulkkinen et al., 2018). In contrast, the TBEV-EU subtype which is prevalent in Europe causes milder infections mainly characterized by a biphasic fever with neurological disorders during the second phase, and case/fatality rates of 0.5–2% (Pulkkinen et al., 2018).

TBEV is mainly transmitted by tick bites. The virus has a major reservoir in rodents from which it is transferred during feeding on viraemic animals to specific tick vectors: *Ixodes ricinus* for the European subtype and *Ixodes persulcatus* for the Asian subtypes (Ruzek et al., 2019). After feeding, infected ticks can transmit the virus back to rodents and also to large animals and humans (Salat & Ruzek, 2020).

A second TBEV transmission pathway to humans is via the consumption of raw milk and raw milk products (Lickova et al., 2021; Buczek et al., 2022). When TBEV-infected ticks feed on cows, sheep and goats, the animals can become viraemic and for a few days the virus can be excreted into the milk (Muhd Radzi et al., 2015; Rodrigues et al., 2019). For example, TBEV could be detected for up to 5 days in the blood and for 3–8 days in the milk of goats after infection (Muhd Radzi et al., 2015; Rodrigues et al., 2019). As TBEV infection in goats is largely asymptomatic, it is often difficult to identify animals potentially releasing the virus into the milk (Buczek et al., 2022). In goat raw milk, the virus was described to be stable for up to 24 h at 4 °C and 22 °C (Offerdahl et al., 2016). In another study, infectious TBEV was detectable for up to 25 days in goat raw milk and up to 15 days in unsalted cheese samples after artificial contamination with a high virus dose of about 2 × 10^5^ plaque forming units (PFU)/ml (Rónai & Egyed, 2020). Although TBEV is relatively sensitive to treatment with heat and detergents, it can still retain its infectivity in normal gastric juice for up to 2 h (Muhd Radzi et al., 2015; Rodrigues et al., 2019). After consumption of raw milk by humans, the virus can therefore reach the duodenum without losing its infectivity, causing infection and disease (Balogh et al., 2012). Several outbreaks of TBE after consumption of raw milk or raw milk products have been reported from Hungary, Austria, Estonia, Slovakia, Czech Republic, and Germany (Salat & Ruzek, 2020; Lickova et al., 2021; Buczek et al., 2022). For example, a large outbreak involving 27 patients, which could be linked to consumption of goat raw milk sold in a supermarket, was described in Estonia in 2005 (Kerbo et al., 2005). More recently, smaller TBE outbreaks resulting from consumption of goat raw cheese and goat raw milk in 2016 and 2017, respectively, were reported from Southern Germany (Brockmann et al., 2018).

Although raw milk and cheese have been repeatedly involved in TBEV transmission, detection methods for the virus in these matrices are only rarely described and—to our best knowledge—comparisons of their efficiency have not been systematically assessed so far. However, detection of viruses in food can be difficult e. g. due to high contents of fat or the presence of PCR inhibitors (Schrader et al., 2012). As milk and cheese are not covered by the current ISO method for the detection of viruses in food (ISO, 2019), no standardized detection methods are available so far.

To compare different extraction methods for the detection of TBEV-RNA in food, goat raw milk and goat raw milk cream cheese were artificially contaminated with Langat virus (LGTV), a naturally attenuated TBEV strain (Rodrigues et al., 2019), and virus recovery rates (RR) were assessed. In addition, LGTV-containing test samples were spiked with Mengovirus or human coronavirus (HCoV) 229E to assess their suitability as process controls. The selected methods were also further characterized regarding their detection limits for TBEV. The methods may be useful for screening or surveillance studies, as well as in outbreak investigations.

## Materials and Methods

### Goat Raw Milk and Raw Milk Cream Cheese Samples

Goat raw milk was kindly provided by the Unit Animal Husbandry, Aquaculture and Reference Material (German Federal Institute for Risk Assessment, Berlin, Germany). Goat raw milk cream cheese was purchased at an organic-food market in Berlin, Germany. Until use, sample aliquots were stored at − 20 °C.

### Cells and Viruses

All cell culture reagents and media were purchased from Pan Biotech (Aidenbach, Germany) unless otherwise indicated. LGTV (from Institute for Diagnostic Virology, Friedrich-Loeffler-Institute, Greifswald-Insel Riems, Germany) was propagated in the human lung carcinoma cell line A549 (ATCC® CCL-185™, Manassas, Virginia, USA) which was maintained in Eagle’s Minimal Essential Medium (EMEM, Sigma-Aldrich Chemie GmbH, Taufkirchen, Germany) supplemented with 10% fetal bovine serum (FBS), 1% non-Essential Amino Acids (NEAA), 1% L-glutamine and 1% gentamicin. Briefly, a 90% confluent A549 cell monolayer was inoculated with LGTV and incubated for three days at 37 °C and 5% CO_2_. The LGTV-containing supernatant was collected and aliquots of this LGTV stock were stored at − 80 °C until further use. Mengovirus strain vMC_0_ (kindly provided by Albert Bosch, Enteric Virus Group, University of Barcelona, Spain) was propagated in HeLa-cells (ATCC® CCL-2™) according to the ISO 15216-1 annex E (ISO, 2019). HCoV-229E (ATCC® VR-740) was propagated in Huh-7 cells (kindly provided by Christian Drosten, Charité, Institute of Virology, Berlin, Germany). The cells were maintained at 37 °C and 5% CO_2_ in Dulbecco’s Modified Eagle’s Medium (DMEM) with 1 × NEAA, 2 mM L-glutamine, and 0.1 µg/ml gentamicin (hereafter referred to as complete DMEM) supplemented with 10% FBS. For HCoV-229E propagation, an 80% confluent cell monolayer was incubated with the virus for 1 h at 34.5 °C. Subsequently, the inoculum was removed, complete DMEM supplemented with 5% FBS added and incubated at 37 °C and 5% CO_2_ for 30 h. Thereafter, the culture flask was frozen once at -80 °C and thawed at 37 °C. Cell debris was removed by centrifugation at 1000 × *g* for 20 min at 4 °C. The supernatant (“HCoV-229E stock”) was stored at − 80 °C until use.

### LGTV and HCoV-229E Titration Using Plaque Assay

For titration of LGTV stock infectivity, A549 cells were seeded into 6-well plates (VWR International, Darmstadt, Germany) at a density of 300,000 cells/well and were incubated for one day. Thereafter, the cells were inoculated with duplicates of a tenfold serial virus dilution (1 ml/well) in MEM at 37 °C and 5% CO_2_. After 3 h, the inoculum was removed and the cell monolayer was covered with 3 ml overlay medium (previously held in a water bath at 40 °C). The overlay medium contained 1% agarose (final concentration) in 1 × EMEM without phenol red (stock solution 2 ×) and supplemented with 10% FBS, 1% NEAA, 1% l-glutamine and 1% gentamicin. The plates were incubated at 37 °C and 5% CO_2_ for 72 h and cells were thereafter fixed with 10% paraformaldehyde (Carl Roth, Karlsruhe, German) overnight at 4 °C. Subsequently, the solution and solid overlay was removed and 1% crystal violet (Merck, Darmstadt, Germany) solution was added. After washing, plaques were manually enumerated and a virus titer of 7 × 10^5^ PFU/ml was determined for the LGTV-stock. For titration of HCoV-229E stock infectivity, Huh-7 cells (6.25 × 10^5^/well) were seeded in 6-well plates in complete DMEM with 10% FBS for 24 h. Subsequently, medium was removed, 500 µl of tenfold virus sample dilutions in DMEM were added to the wells and incubated for 1 h at 34.5 °C and 5% CO_2_ while manually rocking the plates every 10–15 min. After removing the inoculum, the cell monolayer was covered with 3 ml of the same overlay medium as described for the LGTV plaque assay. The cells were fixed at 4 °C for 1 day after adding 1 ml 10% paraformaldehyde solution (Carl Roth, Karlsruhe, German) to the agarose plug. Thereafter, paraformaldehyde was aspirated and discarded, the agarose plug removed and cells stained with 1% crystal violet. Cells were washed with water, dried, and stored in the dark until plaques were manually counted. A virus titer of 2.3 × 10^6^ PFU/ml was determined for the HCoV-229E stock.

### Mengovirus Titration Using 50% Tissue Culture Infectious Dose (TCID_50_) Assay

HeLa-cells were seeded in 96‐well plates and grown under conditions as described (ISO, 2019). Thereafter, the cells were washed two times with phosphate-buffered saline (PBS) and infected with tenfold dilution series of the Mengovirus in medium without additives. After incubation at 37 °C and 5% CO_2_ for 1 h, the virus dilutions were removed and replaced with medium with additives (ISO, 2019). The plates were incubated at 37 °C and 5% CO_2_ until a complete cytopathogenic effect (CPE) was visible in the wells with less diluted virus. The Spearman and Kärber algorithm (Kärber, 1931; Spearman, 1908) was used for the calculation of the TCID_50_. The used Mengovirus stock contained 10^6^ TCID_50_/ml.

### Generation of Artificially Contaminated Food Samples

For generation of artificially contaminated samples, 4 µl of LGTV (7 × 10^5^ PFU/ml) along with 10 µl of Mengovirus (10^6^ TCID_50_/ml) or 35 µl HCoV-229E (2.3 × 10^6^ PFU/ml) as process control were added to 140 µl goat raw milk or to 0.14 g or 0.1 g (method 4 and method 5, respectively) raw goat cream cheese directly before the experiment. As a recovery rate (RR) control (inoculum), 140 µl PBS were mixed with 4 µl of LGTV and either 10 µl of Mengovirus or 35 µl HCoV-229E, respectively.

### Extraction Procedures from Goat Raw Milk

**Fig. 1:**
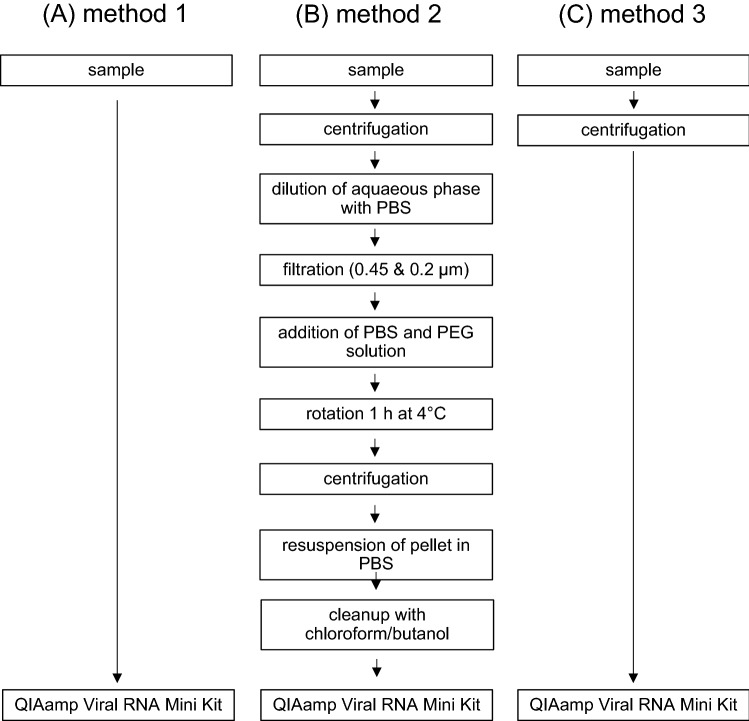
Schematic illustration of the extraction methods tested for goat raw milk. **A** Method 1, based on direct RNA extraction from the sample, **B** method 2, based on PEG precipitation and chloroform/butanol cleanup prior to RNA extraction, **C** method 3, based on sample centrifugation prior to RNA extraction

Three different methods were tested for virus and RNA extraction from goat raw milk, which are schematically shown in Fig. 1A–C. For method 1, viral RNA was directly extracted from 140 µl of the artificially contaminated milk samples using the QIAamp Viral RNA Mini Kit (Qiagen GmbH, Hilden, Germany) according to the manufacturer’s instructions. The RNA was eluted with 60 µl AVE buffer. Method 2 is based on a polyethyleneglycol (PEG) precipitation protocol essentially described in ISO 15216-2 (ISO, 2019). Briefly, 2 ml of the contaminated raw milk sample was centrifuged for 5 min and 6000×*g* at 4 °C. The aqueous interphase was collected and an equal amount of PBS was added. The solution was thereafter filtrated using 0.45 und 0.2 µm filters, and the volume of the filtrate was adjusted to 40 ml with PBS (pH 7.0–7.3). Subsequently, 10 ml of a 10% (w/v) PEG/NaCl-solution was added, followed by incubation on a rotator at 4 °C for 1 h. Thereafter, the solution was centrifuged at 10,000×*g* for 30 min at 4 °C, and the supernatant was discarded. The precipitate was resuspended in 500 µl PBS and subsequently 500 µl of a butanol/chloroform (1:1) solution was added, followed by mixing. After centrifugation at 14,000 rpm for 15 min at 4 °C, 140 µl of the aqueous phase was aspirated and viral RNA was extracted with the QIAamp Viral RNA Mini Kit, as described above. For method 3, 140 µl of the contaminated milk sample were centrifuged at 4 °C and 6000×*g* for 10 min. The aqueous interphase was aspirated and 140 µl (120 µl of aqueous interphase filled up with PBS) was used for viral RNA extraction using the QIAamp Viral RNA Mini Kit.

### Extraction Procedures from Goat Raw Milk Cream Cheese

**Fig. 2:**
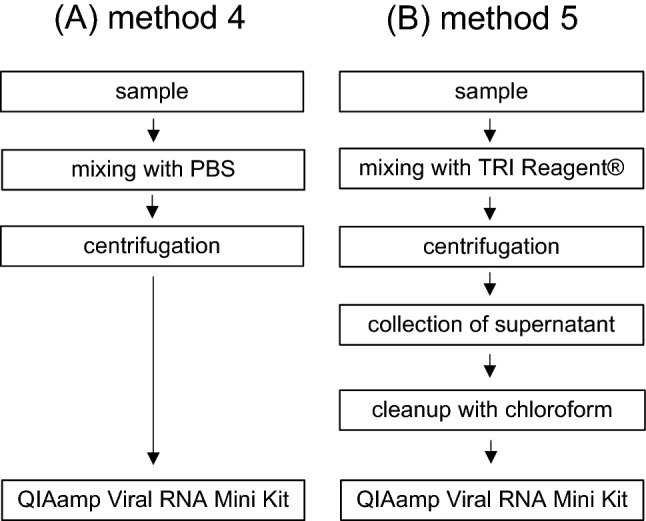
Schematic illustration of the extraction methods tested for goat raw milk cream cheese. **A** Method 4, based on sample centrifugation prior to RNA extraction, **B** method 5, based on extraction with TRI Reagent® and chloroform cleanup prior to RNA extraction

For virus and RNA extraction from goat raw milk cream cheese, two methods were compared which are schematically shown in Fig. 2A and B. In method 4, 0.14 g of contaminated cheese were suspended in 140 µl PBS by vortexing and flicking, resulting in a homogenous solution. The solution was centrifuged at 6000×*g* and 4 °C for 10 min, and the RNA was extracted from 140 µl (120 µl of aqueous interphase filled up with PBS) using the QIAamp Viral RNA Mini Kit. Method 5 is based on an extraction protocol for viral RNA from food with high content of fat and protein (sausages) (Althof et al., 2019). Briefly, 0.1 g contaminated cream cheese were mixed with 350 µl TRI Reagent® (ThermoFisher, Berlin, Germany) and vortexed for 15 s. Thereafter, the mixture was incubated at room temperature for 10 min and subsequently centrifuged at 4 °C and 10,000×*g* for 15 min. The supernatant was transferred into a new tube, 70 µl chloroform were added and vortexed for 15 s followed by a 10 min incubation at RT. After centrifugation at 4 °C and 10,000×*g* for 15 min, the upper aqueous phase (180 µl) was transferred into a new tube and filled up with PBS to 280 µl. Subsequently, RNA was extracted with the QIAamp Viral RNA Mini Kit using the double amount of lysis buffer and reagents before loading to the column as recommended by the manufacturer for 280 µl samples.

### Real-Time Reverse Transcription-PCR (RT-qPCR)

The RNA samples were analyzed for the presence of TBEV-RNA by RT-qPCR as previously described (Klaus et al., 2010). Briefly, the RNA AgPath-ID™ One-Step RT-PCR (ThermoFisher, Berlin, Germany) was used and a 25 µl reaction for each sample was prepared as followed: 0.75 µl RNase-free water was mixed with 12.5 µl RT-PCR buffer, 0.25 µl RT-PCR Enzyme Mix, and 1 µl of each of the two forward primers (5ʹ-ACYAGTCGTGAACGTGTTGAGA-3ʹ and 5ʹ-GTCGTGAACGTGTTGAGAAAAAG-3ʹ) as well as the two reverse primers (5ʹ-CTTWCCTTTCAGRATGGCCTTC-3ʹ and 5ʹ-GACCSCCCCCCTTWCCTTT-3ʹ), respectively. All primers were used at a final concentration of 0.6 µM. Further, 2.5 µl of the probe (5ʹ-FAM-CATCCCCAGCTCTTGTTCTCCTAAG-BHQ1-3ʹ; final concentration 0.25 µM) and 5 µl of RNA sample were added. For the detection of Mengovirus-RNA, the same kit was used with primers and probes as described (Pinto et al., 2009). Briefly, 5.46 µl RNase-free water was mixed with 12.5 µl RT-PCR buffer, 1 µl RT-PCR Enzyme Mix, 0.56 µl reverse primer (5ʹ-GAAGTAACATATAGACAGACGCACAC-3ʹ, 0.89 µM final concentration), 0.32 µl of the forward primer (5ʹ-GCGGGTCCTGCCGAAAGT-3ʹ, 0.51 µM final concentration), and 0.16 µl of the probe (5ʹ-FAM-ATCACATTACTGGCCGAAGC-MGB-3ʹ, 0.256 µM final concentration). HCoV-229E-RNA was detected with the same kit and with primers and probes as described (Corman et al., 2015). Briefly, 3.725 µl RNase-free water, 12.5 µl RT-PCR buffer, 1 µl RT-PCR Enzyme Mix, 1.2 µl of forward (5ʹ-TCYAGAGAGGTKGTTGTTACWAAYCT-3ʹ, 0.48 µM final concentration) and 1.2 µl of reverse primer (5ʹ-CGYTCYTTRCCAGAWATGGCRTA-3ʹ, 0.48 µM final concentration) as well as 0.375 µl of FAM-BHQ1 probe (5ʹ-FAM-TGGCMACTTAATAAGTTTGGIAARGCYGG-BHQ1-3ʹ, 0.15 µM final concentration) were used per reaction. The cycling conditions for all RT-qPCRs were: one cycle of each 45 °C for 10 min and 95 °C for 10 min (reverse transcription and RT inactivation/initial denaturation), followed by 42 cycles of 95 °C for 15 s, 55 °C for 20 s, and 72 °C for 30 s. The reactions were carried out on a QuantStudio 5 PCR-System and analyzed using QuantStudio Design and Analysis 1.5.1 Software (ThermoFisher, Berlin, Germany).

### Recovery Rate Calculation

The recovery rate (RR) for each virus was calculated according to ISO 15216-2 (ISO, 2019). Briefly, a standard curve was generated using the RT-qPCR results of tenfold dilution series of the extracted RNA from the virus stocks. The virus RR was calculated as RR = 10^(ΔCq/m)^ × 100%, where ΔC_q_ = Cq_matrix_ − Cq_inoculum_ (Cq_matrix_ value is that of extracted viral RNA from the matrix, Cq_inoculum_ is that of viral RNA extracted from the inoculum), and *m* is the slope of the inoculum control virus RNA standard curve.

### Statistical Analysis

Differences between the RRs of the three extraction methods from goat raw milk were determined by one-way analysis of variance (ANOVA) with Bonferroni correction (*α* = 0.05). An unpaired Student’s *t*-test (*α* = 0.05) was used to determine differences between RRs of the two virus extraction methods from goat raw milk cream cheese. Statistical analyses were performed using SigmaPlot Version 14 (Systat Software, Inc., San Jose, California).

### Quantification of the Genome Copy Number of LGTV Stock

An LGTV RNA standard was generated and was used for determination of the viral genome copy number per ml of the LGTV stock. Briefly, a gBlocks™ gene fragment of the 5`UTR sequence (nucleotide 52–176 in NCBI reference sequence of LGTV: NC_003690.1) of LGTV (125 bp) was obtained from Integrated DNA Technologies (IDT Coralville, IA, USA) and cloned into pCR4-TOPO using a TOPO TA cloning kit (Thermo Fisher Scientific, Waltham, CA, USA) according to the manufacturer’s instructions. DNA of the resulting plasmid was purified using the QIAGEN Plasmid Mini Kit (Qiagen GmbH, Hilden, Germany), linearized with the restriction enzyme *Pme*I (New England Biolabs, Ipswitch, MA, USA) and used for in vitro transcription with the MEGAscript in vitro transcription system (Thermo Fisher Scientific, Waltham, CA, USA). The transcribed RNA was purified using the Monarch RNA purification kit (New England Biolabs) and quantified by using Qubit™ RNA High Broad Range Assay Kit (ThermoFisher, Berlin, Germany) and the Qubit 4 fluorometer (ThermoFisher, Berlin, Germany). Molecule copy numbers were calculated based on the determined RNA mass and the theoretical molecular weight of the LGTV RNA standard molecule. The LGTV RNA standard stock solution contained 6.38 × 10^12^ copies/µl. RNA was extracted from the LGTV stock (from cell culture as described above) using the QIAamp Viral RNA Mini Kit and subjected to RT-qPCR for TBEV-RNA detection as described above, along with a tenfold dilution series of the LGTV RNA standard stock solution. The LGTV stock had a concentration of 2.35 × 10^9^ genome copies/ml.

### Determination of the Detection Limit

Samples of 140 µl goat raw milk and 0.1 g goat raw milk cream cheese were inoculated with 4 µl of tenfold serially diluted LGTV solutions in water. Contamination levels of 6.7 × 10^7^–6.7 × 10^2^ LGTV genome copies (2 × 10^4^–2 × 10^–1^ PFU) per ml goat raw milk and 9.4 × 10^7^–9.4 × 10^2^ LGTV genome copies (2.8 × 10^4^–2.8 × 10^–1^ PFU) per g goat raw milk cream cheese were used. The milk samples inoculated with the LGTV dilutions were then administered to extraction method 1 and 3, whereas that of cream cheese were tested with method 5. Negative controls consisting of goat raw milk and goat raw milk cream cheese without LGTV inoculation were included in each analysis. Each inoculation level was performed in triplicate for the respective food matrix. RNA was analyzed by RT-qPCR for TBEV-RNA detection as described above. The detection limit was defined as the lowest genome copy number, for which all three subsamples tested positive.

## Results

### Comparison of Extraction Protocols for Goat Raw Milk

In order to compare extraction protocols for goat raw milk, two very different protocols were selected, tested and compared (Fig. 1A and B). The first one is based on a simple RNA isolation procedure directly from the milk samples (method 1). The second is a complex method based on PEG precipitation and chloroform/butanol washing prior to RNA isolation (method 2), which is essentially derived from the ISO method for detection of noroviruses and hepatitis A virus in soft fruits and vegetable samples (ISO, 2019). Mengovirus, which is suggested as process control for the above-mentioned ISO method, and HCoV-229E, which resembles an enveloped RNA virus like TBEV, were tested for their suitability to serve as process controls. For the experiments, goat raw milk samples were artificially contaminated with LGTV along with either Mengovirus or HCoV-229E, respectively. After application of the methods, obtained viral RNA extracts were analyzed by specific RT-qPCR and the RRs were calculated for each condition.

**Fig. 3:**
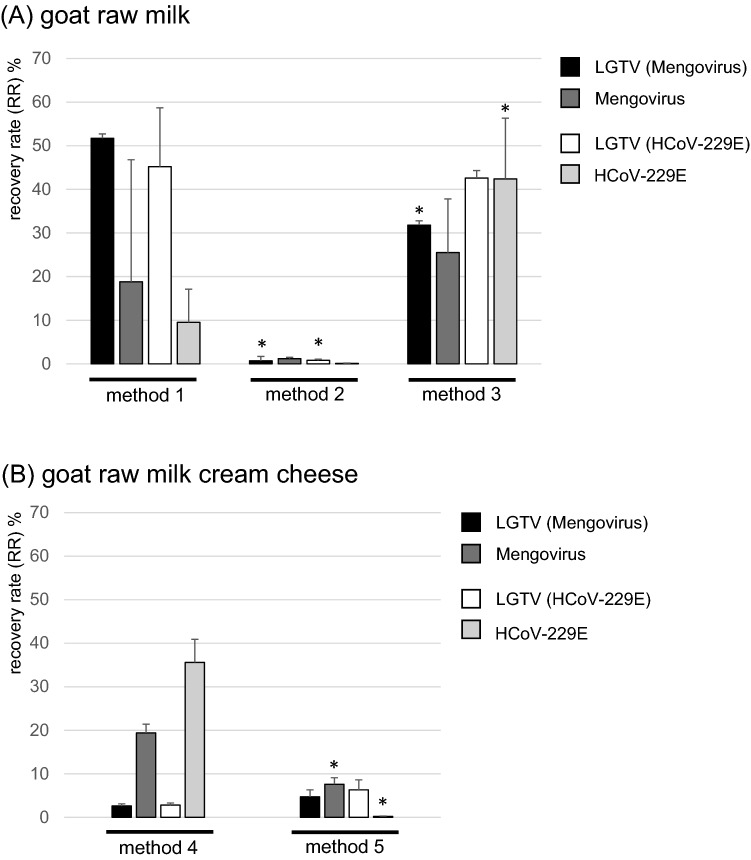
Recovery rates for LGTV-RNA and process control virus-RNA resulting from different extraction methods. **A** Comparison of methods 1–3 applied to artificially contaminated goat raw milk samples. Statistically significant differences (*p* < 0.05) between the RRs of the distinct viruses comparing method 2 with method 1 and method 3 with method 1 (calculated by ANOVA with Bonferroni correction) are indicated with asterisks above the bars. **B** comparison of methods 4 and 5 applied to artificially contaminated goat raw milk cream cheese samples. Statistically significant differences (*p* < 0.05) between the RRs of the distinct viruses comparing both methods (calculated by unpaired Student’s *t* test) are indicated with asterisks above the bars. LGTV (Mengovirus): RR for LGTV-RNA from samples co-contaminated with LGTV and Mengovirus (black bars); Mengovirus: RR for Mengovirus-RNA from samples co-contaminated with LGTV and Mengovirus (dark gray); LGTV (HCoV-229E): RR for LGTV-RNA from samples co-contaminated with LGTV and HCoV-229E (white bars); HCoV-229E: RR for HCoV-229E-RNA from samples co-contaminated with LGTV and HCoV-229E (light gray bars). Mean values (bars) and standard deviations from three independent replicates per condition are shown

As shown in Fig. 3A for method 1 (left panel), RRs of 51.7 ± 8.1% and 45.2 ± 13.5% were determined for LGTV-RNA of goat raw milk samples co-contaminated with Mengovirus or HCoV-229E, respectively. RRs for Mengovirus-RNA and HCoV-229E-RNA were 18.8 ± 28% and 9.5 ± 7.6%, respectively, in these samples. In contrast, for method 2, LGTV-RNA showed RRs of only 0.7 ± 0.2% and 0.8 ± 0.3% for goat raw milk samples co-contaminated with Mengovirus and HCoV-229E, respectively (Fig. 3A, center panel). The RRs for Mengovirus-RNA and HCoV-229E-RNA within these samples were 1.2 ± 0.3% and 0.1 ± 0.07%, respectively. Since higher RRs, but larger standard deviations were observed for method 1, this method was further optimized. In order to remove fat, an additional centrifugation step was added to the procedure, which was further on referred to as method 3 (Fig. 1C). As shown in Fig. 3A (right panel), RRs of 31.8 ± 4.9% and 42.6 ± 1.7% were determined for the LGTV-RNA for goat raw milk samples co-contaminated with Mengovirus and HCoV-229E, respectively. Within the same samples, the RRs for Mengovirus-RNA and HCoV-229E-RNA were 25.5 ± 12.3% and 42.4 ± 13.9%, respectively.

### Comparison of Extraction Protocols for Goat Raw Milk Cream Cheese

For TBEV detection in goat raw milk cream cheese, two different extraction methods were selected, tested and compared (Fig. 2A and B). The first one (method 4) includes only sample dilution and centrifugation prior to RNA isolation, whereas the second one (method 5) is more complex and mainly based on a method for hepatitis E virus detection in sausages, which have a high content of fat and protein (Althof et al., 2019). As shown in Fig. 3B for method 4 (left panel), RRs of 2.6 ± 0.5%, and 2.8 ± 0.5% were determined for LGTV-RNA in samples co-contaminated with Mengovirus and HCoV-229E, respectively. The RRs for Mengovirus-RNA and HCoV-229E-RNA in these samples were 19.4 ± 2% and 35.6 ± 5.3%, respectively. For method 5, LGTV-RNA was recovered by 4.7 ± 1.6% and 6.3 ± 2.3% for samples co-contaminated with Mengovirus and HCoV-229E, respectively (Fig. 3B, right panel). Within these samples, RRs for Mengovirus-RNA and HCoV-229E-RNA were 7.6 ± 1.5% and 0.2 ± 0.1%, respectively.

### Determination of Detection Limits for Selected Methods

Based on the results for RRs of LGTV-RNA presented above, methods 1 and 3 for goat raw milk and method 5 for goat raw milk cream cheese were selected for further characterization regarding determination of detection limits. Samples of both matrices were contaminated in triplicates with tenfold dilution series of LGTV and analyzed for the presence of LGTV-RNA using the selected methods, respectively. As shown in Table 1, the determined detection limit for method 1 was at 6.7 × 10^4^ LGTV genome copies (corresponding to 20 PFU) per ml of goat raw milk and 6.7 × 10^3^ LGTV genome copies (corresponding to 2 PFU) per ml of goat raw milk for method 3. For method 5, the detection limit was at 9.4 × 10^4^ LGTV genome copies (corresponding to 28 PFU) per g of goat raw milk cream cheese.

**Table 1:** Determination of the detection limit of methods for the detection of LGTV-RNA in artificially contaminated goat raw milk and goat raw milk cream cheese

Method (matrix)	Inoculation level (genome copies/ml)	Inoculation level (plaque-forming units/ml)	No. of positive samples/no. of samples tested	Cq value (mean ± SD)
Method 1 (goat raw milk)	6.7 × 10^7^	2 × 10^4^	3/3	22.7 ± 1.2
	6.7 × 10^6^	2 × 10^3^	3/3	26.0 ± 0.4
	6.7 × 10^5^	2 × 10^2^	3/3	29.9 ± 0.8
	6.7 × 10^4^	20	3/3	34.4 ± 0.8
	6.7 × 10^3^	2	0/3	n.d
	6.7 × 10^2^	2 × 10^–1^	0/3	n.d
	–	–	0/3	n.d
Method 3 (goat raw milk)	6.7 × 10^7^	2 × 10^4^	3/3	22.9 ± 0.6
	6.7 × 10^6^	2 × 10^3^	3/3	25.9 ± 0.7
	6.7 × 10^5^	2 × 10^2^	3/3	28.7 ± 0.7
	6.7 × 10^4^	20	3/3	34.3 ± 2.9
	6.7 × 10^3^	2	3/3	38.5 ± 0.4
	6.7 × 10^2^	2 × 10^–1^	0/3	n.d
	–	–	0/3	n.d
Method 5 (goat raw milk cream cheese)	9.4 × 10^7^	2.8 × 10^4^	3/3	24.2 ± 0.4
	9.4 × 10^6^	2.8 × 10^3^	3/3	27.7 ± 0.3
	9.4 × 10^5^	2.8 × 10^2^	3/3	31.8 ± 0.4
	9.4 × 10^4^	28	3/3	36.8 ± 0.8
	9.4 × 10^3^	2.8	0/3	n.d
	9.4 × 10^2^	2.8 × 10^–1^	0/3	n.d
	–	–	0/3	n.d

*n.d.* not detected, – samples without LGTV inoculation

## Discussion

Transmission of TBEV via consumption of goat raw milk and goat raw milk products has been described repeatedly (Brockmann et al., 2018; Chitimia-Dobler et al., 2021; Salat & Ruzek, 2020). However, a systematic comparison of methods for TBEV-RNA extraction in these food matrices has not been published so far. In general, the detection of viruses in food is difficult as the distinct matrices may contain substances which interfere with virus extraction or inhibit the PCR (Schrader et al., 2012; Stals et al., 2012). Therefore, in the present study, we aimed to develop and compare sensitive and reliable methods for this purpose. The selected methods are in part based on protocols previously published for other viruses in different food matrices. An appropriate process control, which represents an important prerequisite for a reliable virus detection method in food (D’Agostino et al., 2011), should also be developed for the method.

For our method comparison, we used raw milk and raw milk cream cheese samples representing typical matrices in foodborne outbreaks, which were artificially contaminated with LGTV. This virus strain resembles all features of TBEV, but is naturally attenuated, thus allowing its use under BSL-2 conditions (Rodrigues et al., 2019). LGTV could be efficiently cultivated and titrated in our study, which made it possible to develop a well-controlled contamination system and which could be used for future comparisons of methods for TBEV detection in food. The system enabled the selection of reliable detection methods for the intended purpose, however, future validations of the methods with naturally contaminated samples, which were not available in our study, would be desirable.

For virus extraction from goat raw milk samples, we first compared a simple RNA extraction method directly from milk (method 1) with a complex elution/concentration method involving PEG precipitation and chloroform/butanol cleanup (method 2). PEG-based precipitation has already been successfully used for TBEV concentration from cell culture supernatant (Füzik et al., 2018). In general, elution/concentration methods have been described to be applicable to a wide range of food types, including those with high fat, protein and carbohydrate contents, which may impact the recovery of microorganisms and their genomic material (Stals et al., 2012). Milk contains those components and it has been shown that milk components can interfere with the recovery of bacteriophage MS2 (Yavarmanesh et al., 2010, 2013). However, our method comparison clearly showed that the RRs for LGTV-RNA in goat raw milk using method 2 were very low. In contrast, the simple and direct RNA isolation of method 1 yielded in high RRs, indicating that the raw milk components do not interfere with RNA extraction and RT-qPCR. In line with this, TBEV has been successful detected in milk of experimentally TBEV-infected goats using a similar protocol with direct RNA extraction from milk (Balogh et al., 2012). However, we identified a high variability of the RRs for LGTV-RNA and the process control viruses using method 1, which we thought could be minimized by the removal of fat (method 3), as it was already described for naturally contaminated milk samples from cattle (Paulsen et al.,^ 2019^). As a result, method 1 and method 3, showing mean RR of 45.2–51.7% and 31.8–42.6%, respectively, were selected for further characterization.

For goat raw milk cream cheese, an easy-to-perform method using only dilution and centrifugation prior to RNA isolation (method 4) was compared with a more complex method using TRI Reagent® extraction and chloroform cleanup (method 5). It was previously shown that the latter method is most suitable for analysis of sausages (Szabo et al., 2015), which have—as it was also expected for cream cheese—a high content of fat and protein. As a result, method 4 showed large differences between the RRs of LGTV-RNA and that of the process control viruses. In addition, method 5 resulted in slightly higher LGTV-RNA-RRs (4.7–6.3%) as compared to method 4 (2.6–2.8%). However, these RRs are considerably lower compared to those determined for goat raw milk, most probably reflecting the more complex composition of the cream cheese matrix. Nevertheless, they are clearly above the RR of 1%, which is considered acceptable according to the ISO method for norovirus and HAV in different food matrices (ISO, 2019).

In order to characterize the most promising methods 1, 3 and 5 further, their detection limits were determined using artificial contamination of the matrices with defined virus dilution series. The lowest detection limits for goat raw milk showed method 3 with 6.7 × 10^3^ LGTV genome copies/ml, and for goat raw milk cream cheese method 5 with 9.4 × 10^4^ LGTV genome copies/g. To our best knowledge, no data on detection limits have been published for other TBEV detection methods, but comparable values have been described for noroviruses in those products, e.g. for genogroup I norovirus 10^5^ genome copies/ml milk and 10^5^ genome copies/g cheese, and for genogroup II norovirus 10^3^/ml milk and 10^3^ genome copies/g cheese (Hennechart-Collette et al., 2017). Taking into account, that in milk samples from naturally infected goats relatively high TBEV loads around 1.88 × 10^5^ RNA copies/ml can be detected (Hudopisk et al., 2013), the detection limits of our selected methods seem to be sufficient for the intended purpose. However, to bring down the limit of detection even more, further optimizations for the cream cheese method are desirable in future.

With regard to the development of standardized methods, inclusion of internal process controls is crucial to monitor the efficiency of all analytical steps (D’Agostino et al., 2011; Hennechart-Collette et al., 2015). Ideally, process control viruses should have morphological and physicochemical properties similar to the target virus and provide comparable extraction efficiency (Lees, 2010). HCoV-229E is an enveloped single-stranded RNA virus like TBEV and might therefore reflect its characteristics well. In contrast, Mengovirus is a non-enveloped RNA virus. However, it is currently widely used as process control for other foodborne viruses and therefore well established in many analytical laboratories (ISO, 2019). For this reason, we have compared the RRs of both viruses along with that of LGTV. For some of the methods (e.g. method 1, Fig. 3A left panel; or method 4, Fig. 3B left panel), large differences were evident between the RRs of LGTV compared to HCoV-229E or Mengovirus, for unknown reasons. However, for the selected method 3, similar RRs were found for all three viruses (Fig. 3A, right panel), indicating that for this procedure both viruses are suitable as internal process controls. For method 5, only Mengovirus showed similar RRs compared to those of LGTV, whereas HCoV-229E had very low RRs (Fig. 3B, right panel), indicating that only Mengovirus can be used for this method. Taken together, we therefore suggest to generally use Mengovirus as internal process control for methods 3 and 5, since it shows similar RRs like LGTV. In addition, laboratory handling might be easier with this virus as compared to the human-pathogenic HCoV-229E.

In conclusion, we were able to establish suitable extraction methods for TBEV-RNA detection in goat raw milk (method 3) and goat raw milk cream cheese (method 5). Mengovirus can be recommended as an internal process control for monitoring the whole analytical process. Future studies should focus on the evaluation of the methods with naturally contaminated field samples. The developed methods can be useful for screening or surveillance studies, as well as in outbreak investigations.

## Data Availability

Additional data can be retrieved upon request by R.J. (Reimar.Johne@bfr.bund.de).
